# Supplementary material for: Effects of Environmental and Electric Perturbations on the pKa of Thioredoxin Cysteine 35: A Computational Study
Source: Molecules. 2022 Sep 30;27(19):6454. doi: 10.3390/molecules27196454 (PMC9570579; doi:10.3390/molecules27196454)
Supplement: Supplementary file 1 [file molecules-27-06454-s001.zip › molecules-1869748-supplementary.pdf]

1. PCA of α2-helix and eigenvector components analysis

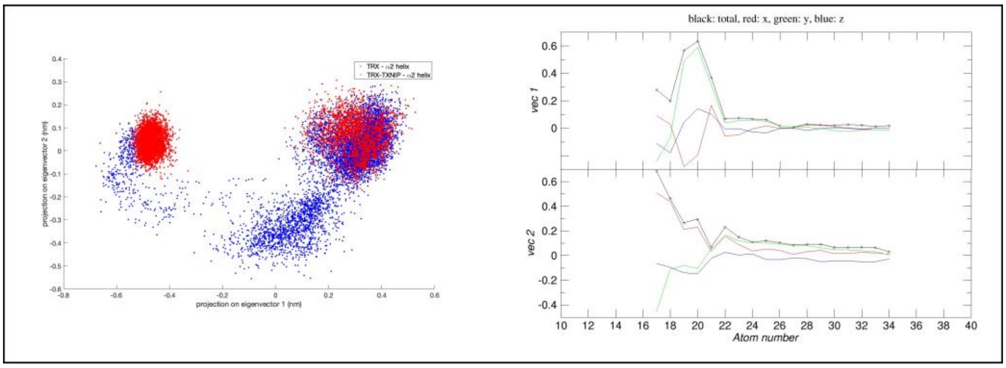

Figure S1. (*left*) PCA for the C-α of α2-helix of TRX (residue 32-49); (*right*) eigenvector components analysis for C-α of α2-helix of TRX.

In the selection of C-α atoms of TRX, we excluded N- and C- terminal residues. For the 77 total C-α analyzed, atomic index from 17 to 34 corresponds to the C-α of the helix and, in particular, index from 17 to 20 are referred to Cys 32 - Gly 33 - Pro 34 - Cys 35 of the active site motif.
